# Caregiver burden and coping strategies in caregivers of older patients with stroke

**DOI:** 10.1186/s40359-021-00556-z

**Published:** 2021-04-01

**Authors:** Azar Kazemi, Jalil Azimian, Maryam Mafi, Kelly-Ann Allen, Seyedeh Ameneh Motalebi

**Affiliations:** 1 Student Research Committee, School of Nursing and Midwifery, Qazvin University of Medical Sciences, Qazvin, Iran; 2 School of Nursing and Midwifery, Qazvin University of Medical Sciences, Qazvin, Iran; 3 Faculty of Education, Monash University, Clayton, Australia; 4 The Centre for Wellbeing Science, Melbourne Graduate School of Education, University of Melbourne, Parkville, Australia; 5 Social Determinants of Health Research Center, Research Institute for Prevention of Non-Communicable Diseases, Qazvin University of Medical Sciences, Qazvin, Iran

**Keywords:** Aged, Stroke, Caregiver, Burden of care, Coping strategies

## Abstract

**Background:**

Coping strategies play a key role in modulating the physical and psychological burden on caregivers of stroke patients. The present study aimed to determine the relationship between the severity of burden of care and coping strategies amongst a sample of Iranian caregivers of older stroke patients. It also aimed to examine the differences of coping strategies used by male and female caregivers.

**Methods:**

A total of 110 caregivers of older patients who previously had a stroke participated in this descriptive and cross-sectional study. The Zarit Burden Interview and Lazarus coping strategies questionnaires were used for data collection. Questionnaires were completed by the caregivers, who were selected using convenience sampling. The collected data were analyzed using Pearson's correlations and independent t-tests.

**Results:**

The mean age of participants was 32.09 ± 8.70 years. The majority of the caregivers sampled reported mild to moderate (n = 74, 67.3%) burden. The most commonly used coping strategies reported were positive reappraisal and seeking social support. Results of the independent t-test showed that male caregivers used the positive reappraisal strategy (t(110) = 2.76; p = 0.007) and accepting responsibility (t(110) = 2.26; p = 0.026) significantly more than female caregivers. Pearson’s correlations showed a significant positive correlation between caregiver burden and emotional-focused strategies, including escaping (r = 0.245, p = 0.010) and distancing (r = 0.204, p = 0.032).

**Conclusions:**

Caregivers with higher burden of care used more negative coping strategies, such as escape-avoidance and distancing. In order to encourage caregivers to utilize effective coping skills, appropriate programs should be designed and implemented to support caregivers. Use of effective coping skills to reduce the level of personal burden can improve caregiver physical health and psychological well-being.

**Supplementary Information:**

The online version contains supplementary material available at 10.1186/s40359-021-00556-z.

## Background

The world’s older population is projected to continue to grow at an unprecedented rate due to improvement in life expectancy and health care techniques [1]. Aging is a period of life in which older people are exposed to potential threats such as chronic conditions, loneliness, isolation, lack of social support, and a decline in independence [2, 3]. Additionally, both physical and psychological (e.g., dementia) chronic diseases tend to become more common with age [3]. Aging is a well-known risk factor for the increase of multiple chronic diseases, including cardiovascular disease, stroke, cancer, osteoarthritis, and dementia [4]. Approximately 80% of older adults have at least one chronic disease; the cost and duration of treatment for these diseases is 20–30 times higher than acute illnesses [2].

One of the major health problems among older adults is stroke, which is the third leading cause of death in the world after cardiovascular diseases and cancer [5, 6]. Stroke is one of the most severe neurological disorders which results from a decrease in cerebral blood flow in certain parts of the brain due to vascular injuries [7]. It is one of the most debilitating neurological conditions that cause chronic and severe disabilities [8, 9]. Stroke patients are very often dependent on their families for their physical and emotional needs after discharge from the hospital [10]. Given that patients often need long-term care, the role of home caregivers is critically important [11].

Essential caregiving for stroke survivors plays an important role in the recovery process, and in preventing additional strokes and improving patients’ overall health [12]. Informal, or home caregivers are critical for long-term care and are often composed of family members, friends, and relatives [13]. Informal caregivers are defined as individuals who provide some type of unpaid, ongoing assistance with activities of daily living or instrumental activities of daily living for individuals with a chronic illness or disability [14]. Oftentimes, one individual serves as the primary caregiver and is assigned to be primarily responsible for most of the physical care and supervision [15]. Caregiving is a difficult task, particularly for untrained primary caregivers who are taking care of an individual with serious, chronic health problems [16]. Unsurprisingly, caregiver stress is common and is caused by the ongoing emotional and physical strain of caregiving [17].

Caregiver burden can be defined as the strain that is experienced by a person who cares for a chronically ill, disabled, or older family member [18]. The burden of care is used to describe the side effects of care that are extremely problematic for the patients and their families [19]. It is a multidimensional response to physical, psychological, emotional, social, and financial stressors associated with the caregiving experience [20, 21]. Caregivers are hidden patients who, as a result of their involvement with caregiving responsibilities, may not be able or eager to seek care for their own health needs [22]. Caregiver burden and strain have been associated with increased health-risk behaviors (such as smoking) and higher rates of drug use [23]. Caregiver mental health can be even more at risk when caregivers perceive that the patient's care needs exceed their caregiving capabilities [10]. Most studies of family caregivers of stroke survivors have reported that caregiving had negative impacts on the caregivers’ health and well-being [24, 25]. Due to the abrupt onset of disability and the chronic nature of stroke recovery, caring for a stroke survivor has been found to have a negative impact on the physical, mental, and psychological health of caregivers [26, 27]. Primary caregivers of stroke patients tend to report more somatic and depressive symptoms, sleep disorders, stress and social isolation than general population [28].

Support is needed to enable informal caregivers to continue in their role as long as possible, without compromising their physical or mental health. Effective and adaptive coping strategies may play a protective role in reducing the caregiver's distress [29]. According to Lazarus and Folkman et al. [30], coping is a process that addresses how people respond and act both when experiencing stress and when the level of exposure to stress rises. Coping strategies are the cognitive and behavioral efforts of individuals to interpret and overcome problems and challenges [31, 32]. It has been proposed that females and males use different coping strategies to deal with stressors [33]. Previous studies about gender differences in the use of coping strategies showed that differences in preferred coping strategies existed [34–36].

Coping strategies have been conceptualized in a variety of ways in the literature, however more broadly, they have been considered to fall into two main categories: problem-focused and emotion-focused [37]. Problem-focused coping strategies aim to change the situation and take control of the source of stress. They involve evaluating the source of stress and actively considering and implementing potential solutions to reduce the aversive effects of the stressor [38, 39]. On the other hand, emotion-based coping involves emotional response to stressors. Emotion-focused coping strategies can also entail enlisting emotional support from others [40].

Eight ways of coping are identified based on the ways of coping (WOC) Questionnaire by the Folkman and Lazarus. These include: Confrontive Coping and Planful Problem-Solving classified under problem-focused coping, and Distancing, Self-Controlling, Accepting Responsibility, Positive Reappraisals, and Escape-Avoidance considered to be emotion-focused coping. The coping strategy of Seeking Social Support functions as both problem and emotion-focused coping.

Confrontive coping refers to the aggressive effort to modify stressful situations while planful problem solving involves analytic approach to providing solutions to problems [41]. Detaching oneself from stressful situations is described as distancing and this strategy may be common for caregivers who do not feel they have the coping resources to face a particular scenario they find confronting [30]. Distancing relates to denial, distracting, or detaching [42]. This may be seen when a family member is diagnosed with a stroke where a caregiver may turn to distractions to avoid acknowledging the problem.

Self-controlling is the individual’s effort to manage their own emotions and actions. Accepting responsibility involves acknowledging one’s contribution to the problem and doing the right thing [43, 44]. For caregivers this can be considered an adaptive and useful coping strategy. Accepting the situation and acknowledging they are in control of how they can respond and cope to it can be an important part of managing news of a stroke. This may assist with managing negative psychological sequalae that may result to death-related depression or anxiety as well as preparatory grief and loss [45–48].

Another coping strategy that is focused on positively interpreting situations is positive reappraisal, wherein thinking about stressful event may be re-framed to be considered as benign, valuable, or beneficial [43, 44]. It is often associated with personal growth and some religious dimensions [32]. For caregivers, positive appraisal may involve considering stroke as unavoidable or inevitable. They may consider the situation as fortunate in that it did not result in death. Unlike hope and optimism [49–53], reappraisal-type strategies are often used in cognitive behaviour therapy [54] and could be seen as a productive and rational coping strategy.

Escape-avoidance is described as the thoughts and behavioral efforts to escape stressful situations or problems [47]. This may involve actively avoiding the problem or withdrawing from others or the situation causing stress. It can also involve wishful thinking. Escape-avoidance and distancing can have negative implications on a person being cared for [42]. A caregiver who withdraws support and care can either physically or psychology, place people with a chronic disease or injury at great risk.

Seeking social support is a commonly used adaptive coping strategy which refers to sharing feelings and thoughts, or seeking care, resources, or emotional support from others [55, 56]. Caregivers may seek social support from family or friends, or elicit information from professionals which can be a form of social support as well [57]. Social support and intergenerational contact has been found to be essential for health outcomes in older adults, ageing, and ageism [58].

Despite the high prevalence rates of stroke in Iran [59] and caregiving responsibilities of family members [60] as well as the reported negative impacts of caregiving on caregiver health status, psychological problems, and the quality of life of caregivers [61], only few studies have examined the coping strategies adopted by caregivers of stroke patients in Iran. Therefore, the aim of this study was to determine the relationships between caregiving burden and coping strategies used by a group of caregivers of older patients with a history of having a stroke to better understand this area of healthcare.

We aimed to answer the following questions:

- What is the level of care burden in caregivers of older patients with a stroke?
- What are the most common coping strategies used by caregivers of older patients with a stroke?
- What kind of relationship is there between care burden and coping strategies in caregivers of older patients with a stroke?
- Do coping strategies used differ based on the gender of the caregiver?

## Methods

This cross-sectional and correlational study was conducted on 110 caregivers of older patients with a history of stroke. The convenience sampling method was used for selecting the representative sample. To identify the caregivers, medical files of stroke patients hospitalized in the Valiasr hospital in the six-month period before the start of the study were extracted and reviewed. Contact information for the caregivers of stroke patients was extracted from the files, and they were then called for the initial screening. Valiasr hospital is the only hospital in Zanjan, Iran that admits stroke patients.

Inclusion criteria for primary caregivers included a willingness to participate, aged 18 years old or over, being able to communicate, having at least a primary level education, being the principal caregiver for a minimum of 1 month, not being paid for the care provided, and having a family relationship with the older patient. Caregivers who were caring for multiple patients were not considered for inclusion in this study. Older patients who were included in the study are 60 years old and above, have been diagnosed with stroke, and have a family caregiver. Those who met the inclusion criteria based on their report were invited to come to the rehabilitation centers or clinic of neurology, depending on the region where they resided. At the time of the phone call, an explanation about the study purpose and procedures were provided to the potential participants.

It was determined that 100 participants were needed to find a correlation coefficient (r = 0.25) between care burden and coping strategies, extracted from a similar study by Alnazly et al. [34], at an alpha level of 0.05, and 90% power. By considering a 10% nonresponsive rate, a sample size of 110 caregivers was finalized for this study.

Data collection was carried out from December 2017 to May 2018. The questionnaires were completed by the primary caregivers of the older patients.

### Instruments

Zarit Burden Interview (ZBI), Lazarus coping strategies questionnaires, and demographic checklists were used to gather the data.

The ZBI questionnaire was used to measure caregiving burden. It is the most widely used instrument to assess perceived caregiving burden in clinical and research settings [62], which measures the physical, emotional-psychological, social, and economic impacts of caregiving. This questionnaire is consists of 22 items and is scored on a five-point Likert scale where 0 = “never” and 4 = “nearly always.” The total ZBI score ranges from 0 to 88 points. Higher scores indicate greater burden [63]. The ZBI is a validated instrument for Iranian populations. Navidian et al. [64] have culturally adjusted this scale; its congruent validity was confirmed with a positive correlation with the Hamilton anxiety scale (r = 0.89) and Beck depression inventory (r = 0.67).

The Lazarus coping strategies questionnaire [30] consists of 66 questions about coping with a stressful situation. Responses are given on a 4-point Likert scale, ranging from 0 = “does not apply and/or not used” to 3 = “used a great deal.” It measures 8 subscales consisting of confrontive coping (6 questions), distancing (6 questions), self-controlling (7 questions), seeking social support (6 questions), accepting responsibility (4 questions), escape-avoidance (8 questions), problem-solving (6 questions), and positive reappraisal (7 questions). The remaining questions are distractor items. Higher scores in each subscale indicate greater use of that particular coping strategy [65]. The Lazarus coping strategies questionnaire was validated in Iranian students [66]. Internal consistency of this questionnaire is high (Cronbach's alpha = 0.85) as confirmed by Ramzi et al. [67].

Demographic characteristics for caregivers included age, gender, marital status, educational level, financial status, job, and history of chronic illnesses. Demographic characteristics for the older patients included age, gender, marital status, and level of education.

### Ethical considerations

All stages of the study were based on the Provisions of the Declaration of Helsinki of 1975. After giving information about the purpose and procedure of the study and prior to completing the questionnaires, written informed consent forms were signed by all the caregivers. The study was approved by the Ethics Committee of Qazvin University of Medical Sciences, Qazvin, Iran (IR.QUMS.REC. 1396.371).

### Statistical analysis

All statistical analyses were conducted using the Statistical Package for Social Science version 19 (SPSS IBM V.19, New York). Descriptive statistics were used to report the characteristics of the caregivers and the severity of burden of care. Continuous variables were presented as mean and standard deviations and categorical data were reported using frequencies and percentages.

Pearson’s correlations were used to examine the association between caregiver burden and coping strategies. Differences in the mean scores of coping strategies scales for gender were analyzed using independent t-tests. The statistical significance level was set at p < 0.05.

## Results

A total of 110 caregivers with mean age of 32.09 ± 8.80 years participated in this descriptive and cross-sectional study. Caregivers were predominately women (n = 77, 70.0%), married (n = 75, 68.2%), unemployed (n = 76, 79.1%), and had children (n = 67, 60.9%). Nearly half of the sample had a diploma or academic education (n = 56, 50.9%) and had a self-reported middle‑range income (n = 60, 54.5%). Nearly 90% of the caregivers reported not having any chronic diseases.

The mean age of care recipients was 69.91 ± 10.50 years old. More than half were female (n = 57, 51.8%), the majority were married (n = 82, 74.5%), and illiterate (n = 82, 74.5%; see Table 1).

**Table 1:** Sociodemographic characteristics of caregivers of older patients with stroke

Demographic characteristics	n	%
Sex		
Female	77	70.0
Male	33	30.0
Marital status		
Married	75	68.2
Single	35	31.8
Job		
Unemployed	76	79.1
Retired	4	3.6
Employed	30	27.3
Children		
0	43	39.1
1	29	26.4
≥ 2	38	34.5
Education level		
Illiterate	6	5.5
Under diploma	48	43.6
Diploma or higher	56	50.9
Financial status		
Low	33	30.0
Middle	60	54.5
High	17	15.5
History of chronic illnesses		
Yes	12	10.9
No	98	89.1

The mean of caregiving burden was 32.80 ± 11.97. The majority of the caregivers suffered from mild (n = 58, 52.7%) to moderate (n = 51, 46.4%) burden and just one caregiver (0.9%) was under severe burden. The results showed that the most common coping strategies used were positive reappraisal (15.16 ± 5.18) and seeking social support (8.58 ± 2.90), and the least were direct confronting (6.58 ± 2.70) and escape-avoidance (14.37 ± 4.98). The greatest caregiver burden was physical burden (15.49 ± 5.06) and the least was social burden (4.68 ± 3.56) (see Table 2).

**Table 2:** Means and standard deviations of the subscales of burden and coping strategies

Variable	Subscales	Mean ± SD	Mean/number of items
Burden care	Physical	15.49 ± 5.06	1.70
	Emotional	9.83 ± 4.60	1.40
	Economic	2.79 ± 2.20	1.39
	Social	4.68 ± 3.56	1.17
	Total	32.79 ± 15.42	19.59
Coping strategies	Positive reappraisal	15.16 ± 5.18	1.51
	Seeking social support	8.58 ± 2.90	1.43
	Problem-solving	12.45 ± 4.28	1.38
	Self-controlling	12.28 ± 4.13	1.36
	Accepting responsibility	7.90 ± 2.94	1.31
	Distancing	8.15 ± 3.14	1.16
	Confronting	6.85 ± 2.70	1.14
	Escape-avoidance	14.37 ± 4.98	1.10
	Total	85.75 ± 19.25	10.39

**Fig. 1:**
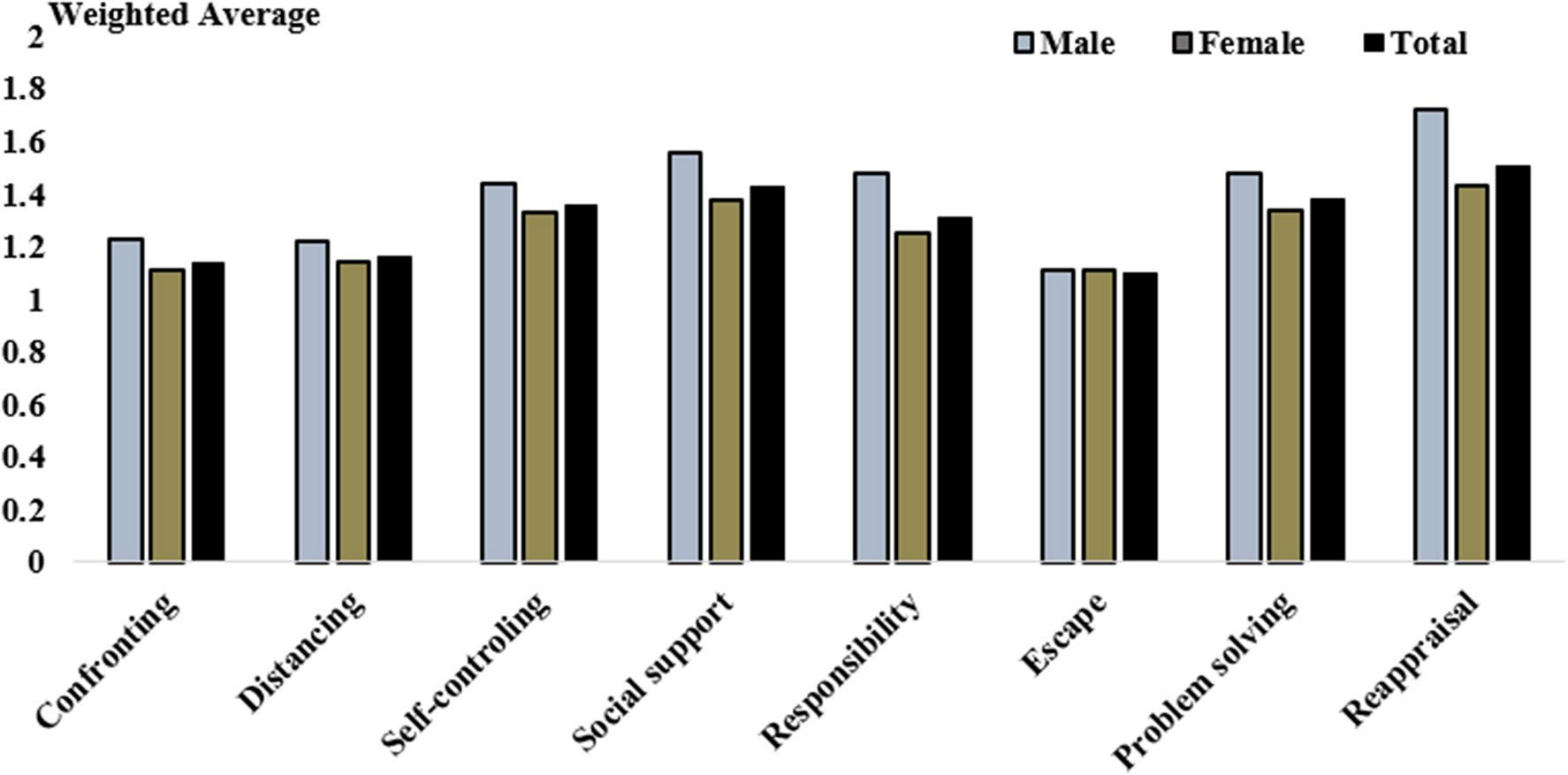
Weighted average of subscales of coping strategies among caregivers of elderly patients with stroke

Figure 1 shows the average range of coping strategies used amongst caregivers of older patients with a history of stroke based on gender. Results of the independent t-test showed that male caregivers used the positive reappraisal strategy (t(108) = 2.76; p = 0.007) and accepting responsibility (t(108) = 2.26; p = 0.026) significantly more than female caregivers.

The information displayed in Table 3 shows that the burden of care was significantly and positively correlated with use of the escape-avoidance coping strategy (r = 0.245, p = 0.010) and distancing coping strategy (r = 0.204, p = 0.032).

**Table 3:** Associations between caregiving burden and coping strategies and its subscales

	Reappraisal	Problem solving	Escape	Responsibility	Social support	Self-controlling	Distancing	Confronting	Coping strategy (total)
Caregiving burden									
r	0.183	0.078	**0.245**	0.156	0.165	0.052	**0.204**	0.102	**0.238**
p values	0.055	0.420	**0.010**	0.103	0.085	0.586	**0.032**	0.291	**0.012**

Bold indicates statistically significant associations (p < 0.05)

## Discussion

Coping strategies are common ways for dealing with demands and in situations that are perceived as threatening [68]. Therefore, the present study aimed to determine the associations between caregiving burden and use of coping strategies among caregivers of older patients with a history of stroke. It also sought to determine the differences in coping strategies used by male and female caregivers.

The present study findings revealed that the care burden reported by the majority of caregivers of stroke survivors was mild to moderate. The findings are partially supported by Kumar et al. [16] who reported that 63% of caregivers of patients with stroke suffered from mild to moderate care burden. However, Rawat et al. [69] reported that more than half (56.67%) of stroke caregivers felt exhausted (high/extreme burden). The reason for these different results in the care burden of caregivers of stroke patients may be related to the degree of dependence of the patients for the daily living activities. In this regard, Baumann et al. [70] found that a decrease in patients’ ability to perform daily activities was significantly associated with an increase in the care burden of caregivers of stroke patients.

Based on the results of this study, women compromised a large majority of caregivers. This is consistent with national [71, 72] and international [73–75] studies which have found that most caregivers are women. A possible explanation for this might be that women believe that caregiving is their role and responsibility, as caregiving of children and other family members is often undertaken by women. Furthermore, women are more emotional than men and are willing to sacrifice their social life for their family [72], and they request little assistance from others, even if social support is available [76]. The results of the present study did not find any differences between men and women in the burden of caregiving. Although other studies have not detected any significant relationship between gender and caregiver burden even across different types of burden [77, 78], some previous studies have reported that female caregivers experience more caregiver burden than their male counterparts [74, 79]. This discrepancy may be related to Iranian culture, where women believe that caregiving is their duty and is expected of them from family members and society.

The results showed that caregivers used more positive reappraisal and social support. Likewise, Alnazly [34] showed that caregivers of dialysis patients used more self-control and positive reappraisal. Ayuarno et al. [29] also found that caregivers of patients with Alzheimer’s disease were more likely to use emotion-focused strategies [80]. Similarly, García-Alberca et al. [81] reported that the use of emotion-focused or problem-solving coping strategies is significantly associated with the level of psychological distress in Alzheimer disease caregivers. Papastavrou et al. [82] also found that the type of coping strategy used among caregivers of cancer patients is related to the levels of care burden. Furthermore, coping strategies are typically related to the task/problem that the caregiver is facing [83].

The results of this study showed that male caregivers were more likely to use the strategies of positive reappraisal and accepting responsibility compared to female caregivers. Consistent with the results of this study, Hassan et al. [35] reported that male caregivers of patients with schizophrenia were more likely to use reappraisal coping strategies than their female counterparts. However, Alnazly [34] found that male caregivers of patients undergoing hemodialysis used distancing more than women caregivers. In addition, Suriyamoorthi et al. [36] showed that male caregivers of patients with bipolar disorder used self-distraction and substance use as coping strategies while females used religion and denial. These different results may be related to the type of care needed based on the differences in patients’ illnesses.

Based on the results of the present study, caregivers with higher care burden used more emotion-focused strategies such as escape-avoidance and distancing. The results of many previous studies are in line with this result. Kumar et al. [68] reported that caregivers of stroke patients under greater burden used emotion-focused coping strategies. Abbasi et al. [83] also showed that there was a direct and significant relationship between the use of emotion-focused coping skills and increasing care burden of the caregivers of cancer patients. As caregivers with high burden care did not adopt appropriate coping strategies and tended to employ emotion-focused coping [82, 84]. In other words, increasing the burden of care beyond the caregiver’s ability can result in reliance on less effective emotion-based strategies rather than more effective problem-focused coping strategies and consulting with others [85].

### Limitations

The present study includes a few limitations. This study only focused on caregivers of older patients with a history of stroke. As the literature shows a variety of other coping strategies are used depending on the type of illness present, the findings cannot be extended to caregivers of all older patients. Second, since this study was conducted on Iranian caregivers, it may be difficult to extend the findings to other countries or cultures. Third, convenience sampling was used, which reduces generalizations to all caregivers of older patients with a history of stroke. Fourth, the limited sample size of the male caregivers (N = 33) may not have been sufficient to find other statistical differences across the subscales. Finally, the nature of the study and the type of analyses used limit the findings to associations and not causal influence.

## Conclusion

Caregivers adopted varied types of coping strategies to overcome burden and the adoption of coping strategies was associated with the severity of burden in caregivers of stroke patients. The informal caregivers with higher burden of care used emotion-focused strategies which often do not help in reducing caregiver stress. As such, training programs that teach caregivers efficient coping strategies are needed in order to increase their use of effective and healthy coping strategies. Furthermore, psychosocial support should be provided by governmental and nongovernmental organizations to reduce the care burden of caregivers of older stroke patients.

## Supplementary Information

**Additional file 1:** Caregivers burden and coping strategies. SA Motalebi, Sup. data.

## Data Availability

All data generated or analyzed during this study are included in this published article [supplementary file:SPSS file].

## Abbreviations

WOC: Ways of coping
ZBI: Zarit Burden Interview
